# Hepatocyte-Derived Apoptotic Bodies as Pathological Intercellular Messengers in the Liver

**DOI:** 10.3390/biom16020198

**Published:** 2026-01-28

**Authors:** Moses New-Aaron, Lukman A. Adepoju, Anup Singh Pathania, Kusum K. Kharbanda, Natalia A. Osna

**Affiliations:** 1 Division of Pulmonary, Allergy, Critical Care and Sleep Medicine, Department of Medicine, Emory University School of Medicine, Atlanta, GA 30322, USA; 2 Atlanta Veterans Affairs Health Care System, Decatur, GA 30033, USA; 3 Department of Pharmacology and Experimental Neuroscience, University of Nebraska Medical Center, Omaha, NE 68198, USA; ladepoju@unmc.edu (L.A.A.); anup.pathania@unmc.edu (A.S.P.); 4 Research Service, Veterans Affairs Nebraska-Western Iowa Health Care System, Omaha, NE 68105, USA; kkharbanda@unmc.edu; 5 Department of Internal Medicine, University of Nebraska Medical Center, Omaha, NE 68105, USA; 6 Department of Biochemistry & Molecular Biology, University of Nebraska Medical Center, Omaha, NE 68198, USA

**Keywords:** hepatocyte apoptotic bodies, non-alcoholic steatohepatitis, apoptosis, extracellular vesicles, alcoholic liver disease, intercellular communication

## Abstract

Hepatocyte apoptotic bodies (ApopBDs) are extracellular vesicles formed during hepatocyte apoptosis. Although they were initially recognized as cellular waste and vesicles that clear toxic substances and viral infections in the liver, they are now known to serve as key mediators of intercellular communication that influence key metabolic and immune responses, such as inflammation, regeneration, and fibrosis. While numerous functions of ApopBDs in the liver are emerging, this review will focus on discussing their biogenesis, characterization, and roles in different liver diseases, with an emphasis on intercellular communication with liver-resident cells. The mechanisms of liver injury are convoluted by series of injurious crosstalk between hepatocyte ApopBDs and surviving resident cells. A unique feature of liver injury is a constant cycle of hepatocyte apoptosis, which has been attributed to crosstalk between surviving hepatocytes and their ApopBDs. The progression of liver injury is also affected by the activation of proinflammatory and profibrotic pathways such as TLR9/NLRP3 and JAK-STAT3. Given the expression of hepatocyte-specific molecular signatures on these ApopBDs, their application as diagnostic tools may improve the treatment of liver diseases. Although the science of hepatocyte ApopBDs is fairly recent and still emerging, in-depth understanding of this aspect of liver biology may provide a novel therapeutic option for the progression of liver damage.

## 1. Introduction

Liver injury, a significant global health issue causing nearly 2 million deaths annually, refers to damage to hepatocytes that results in structural and functional impairment of the liver [1]. Hepatocyte apoptosis, a highly controlled form of cell death, is a critical pathological characteristic of liver injury [2]. Hepatocytes account for 80% of liver cellular distribution [3]; therefore, if hepatocyte apoptosis is not properly managed, it can have disproportionate functional consequences for the liver. While apoptosis canonically serves as an adaptive mechanism to eliminate DNA-damaged or virus-infected hepatocytes to maintain liver homeostasis [4], it can become maladaptive when it occurs excessively and for prolonged periods. This maladaptive response alters the liver microenvironment, promoting inflammation, fibrosis, impaired biosynthetic function, and metabolic failure [5].

Both clinical observations and experimental findings have established an association between hepatocyte apoptosis and liver diseases. For example, plasma cytokeratin-18, a biomarker of hepatocyte apoptosis, has been associated with liver inflammation and fibrosis in patients with nonalcoholic steatohepatitis (NASH) [6]. Another study demonstrated that cleavage of caspase 3, an apoptosis-executing protein, plays a critical role in the progression of nonalcoholic fatty liver disease [7]. The interplay among lipid overload, endoplasmic reticulum (ER) stress, death receptors, and stress-induced molecular pathways, including c-Jun N-terminal kinase, C/EBP homologous protein, and the apoptotic marker Bax, has been widely described as the basis for hepatocyte apoptosis in nonalcoholic fatty liver disease [8,9,10]. The reversal of liver disease progression by pharmacological inhibition of hepatocyte apoptosis [9,10,11,12] substantiates the role of hepatocyte apoptosis in non-alcoholic liver disease. Beyond non-alcoholic liver injury, hepatocyte apoptosis also contributes to other forms of liver injury.

In alcoholic steatohepatitis, the induction of hepatocyte apoptosis via immune responses such as neutrophil extracellular traps was found to contribute to liver damage [8]. Mechanistic studies of alcohol associated liver injury have implicated oxidative stress generated by the oxidative metabolism of alcohol in the contribution to hepatocyte apoptosis [9,10]. Moreover, preclinical studies have linked immune activation-induced liver injury to hepatocyte apoptosis [8,11,12,13], and these findings were confirmed in human studies [14,15,16]. Hepatocyte apoptosis has also been implicated in viral hepatitis [3,17,18,19], congenital liver diseases [20,21], drug-induced liver injury [22], autoimmune liver diseases [23,24], cholestatic liver diseases [5,25] and vascular liver diseases [26].

While the direct effects of hepatocyte apoptosis on liver injury are well documented, particularly through hepatocyte depletion, the emergence of hepatocyte apoptotic bodies (ApopBDs)—vesicles formed from apoptotic hepatocytes—has become a notable factor in elucidating liver pathogenesis. Therefore, this review will focus on hepatocyte ApopBDs and their contribution to liver injury through intercellular communication among liver cells. But first, what are hepatocyte ApopBDs? Hepatocyte ApopBDs are large extracellular vesicles, EVs, generated during hepatocyte apoptosis [27]. They actively mediate injurious intercellular communication with various liver cells (Kupffer cells, KCs, hepatic stellate cells, HSCs, hepatocytes, and liver sinusoidal endothelial cells, LSECs), influencing immune responses, fibrosis, and viral propagation.

## 2. Biogenesis Hepatocyte ApopBDs

**Figure 1:**
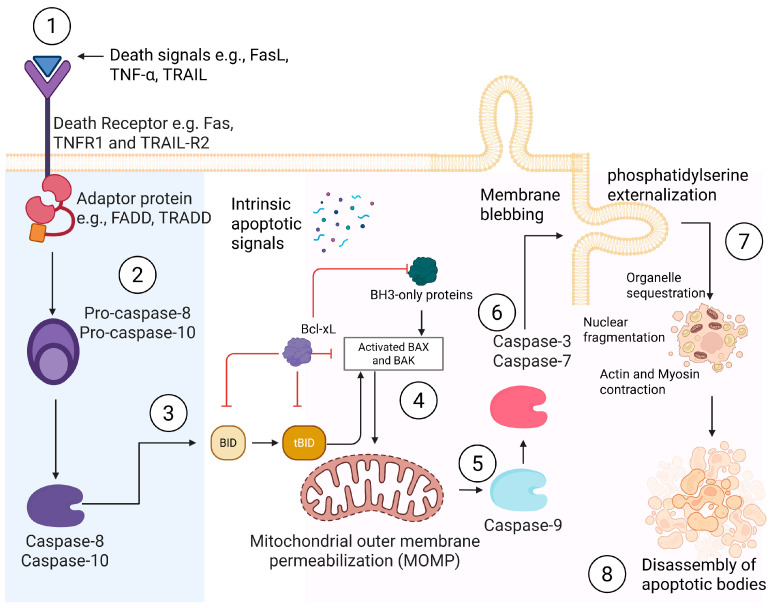
Biogenesis of hepatocyte apoptosis bodies. 1. The interaction between death ligands and their corresponding death receptors initiates the extrinsic apoptotic pathway. 2. The formation of a death-inducing signaling complex occurs when death domains, death effector domains, and procaspases 8 and 10 come together. The continuous transmission of death signals resulting from ligand-receptor interactions increases the concentration of procaspase 8, ultimately leading to its autocleavage. 3. Once active, caspase 8 cleaves Bid into tBid, 4. which in turn activates Bax and Bak, prompting the permeabilization of the mitochondrial outer membrane. Bax and Bak are also activated by intrinsic apoptotic signals detected by BH3-only proteins. 5. The release of mitochondrial intermembrane proteins, such as cytochrome c, interacts with apoptotic protease-activating factor 1 to form an apoptosome, thus initiating the activation of caspase 9. 6. Active caspase 9 subsequently activates caspases 3 and 7, leading to 7. membrane blebbing, organelle sequestration, nuclear fragmentation, and the contraction of actin and myosin, resulting in 8. the formation of ApopBDs.

Understanding the roles of hepatocyte ApopBDs and their contribution to intercellular communication with resident liver cells, both in health and disease, requires a clear understanding of their biogenesis. The formation of ApopBDs begins when hepatocytes are exposed to pro-apoptotic stressors, such as fatty acids, viruses, or alcohol. This process ends in the formation of bioactive membrane-bound vesicles, as described in Figure 1.

### 2.1. Hepatocyte Apoptosis

Unlike cells in other tissues, hepatocytes are particularly vulnerable to injury because of their high metabolic function; therefore, they are highly susceptible to death receptor-mediated apoptosis [28]. Understanding the mechanisms and factors that drive hepatocyte apoptosis is crucial for comprehending the formation of ApopBDs. Apoptosis is activated by either the intrinsic or the extrinsic pathway. The intrinsic pathway depends on mitochondrial changes, whereas death receptors mediate the extrinsic pathway [29]. It is well known that the B cell lymphoma (Bcl)-2 family proteins are key regulators of intrinsic apoptosis [30]. In contrast, death receptors are hallmarks of the extrinsic apoptotic pathway [31]. Susceptibility of hepatocytes to extrinsic apoptosis is partly because hepatocytes constitutively express death receptors [28,32]. However, some studies focusing on free fatty acids [33], alcohol, and HIV [19] have characterized the intrinsic pathway as the driver of liver injury. Whether hepatocyte apoptosis is extrinsically or intrinsically mediated, both pathways, as we will learn in subsequent sections, are executed via the mitochondria. Suffice it to say that mitochondria are the central hub for both intrinsic and extrinsic apoptosis in hepatocytes.

#### 2.1.1. Intrinsic Hepatocyte Apoptotic Pathway

Although intrinsic hepatocyte apoptosis was commonly connected to oxidative [3,34,35] and ER stress [36,37]. Some investigations have linked hepatocyte apoptosis to metabolic products, such as free fatty acids [33] and bile acids [38]. Stress signals trigger the activation of Bcl-2 Homology (BH)-3, a proapoptotic protein, and concurrently degrade Bcl-xL, an antiapoptotic protein, both of which are predominantly localized to the outer mitochondrial membrane [39]. This shift in proapoptotic and anti-apoptotic proteins flags hepatocyte mitochondria for permeabilization. Outer membrane permeabilization is fully executed when BH3 activates proapoptotic cytosolic Bax/Bak proteins after translocation to mitochondria [40]. When the outer membrane of the mitochondria becomes permeable, it releases cytochrome c and other apoptogenic factors. These factors then move from the mitochondria into the cell’s cytoplasm, activating the process of apoptosis by cleaving specific proteins known as caspases 9 and 3/7 [41]. BH3 proteins translate death signals from metabolic products, oxidative stress, and ER stress into mitochondrial events that lead to apoptosis [42].

#### 2.1.2. Extrinsic Hepatocyte Apoptotic Pathway

The involvement of the extrinsic apoptotic pathway in hepatocyte injury cannot be overemphasized. The interaction of death ligands such as Fas Ligand (FasL), Tumor Necrosis Factor, TNF,-α, and TNF-Related Apoptosis-Inducing Ligand, TRAIL, to their corresponding death receptors, including Fas, Tumor Necrosis Factor Receptor (TNFR)-1, and TRAIL receptor 1/2, initiates the process of extrinsic apoptosis [29]. To connect hepatocyte extrinsic apoptosis to liver injury, the synergistic interaction of Fas and TNFR1 was demonstrated in rodents’ alcoholic liver injury [43,44]. Another study showed that the interaction of TNF-α and TNFR1 is a critical factor for hepatocyte apoptosis-mediated acute liver injury [45]. These death ligands are canonically generated in the inflamed hepatic microenvironment by non-parenchymal liver cells, such as activated KCs. The interaction between death ligands and their receptors triggers signals that bring pre-existing death domains close together. This enables the formation of a death-inducing signaling complex through the assembly of primary extrinsic apoptotic initiators. These initiators include Fas-associated death domain protein, tumor necrosis factor receptor type 1-associated death domain protein, death effector domains, and procaspase 8/10 [46]. If assault on the liver continues, the concentration of procaspase 8 rises due to ongoing apoptotic signals from the interaction with death ligand receptors, resulting in the autoproteolytic cleavage of procaspase 8 to form active caspase 8. Unlike the so-called “type 1 cells”, hepatocytes, which are described as “type 2 cells,” do not directly cleave caspase-3/7 via caspase-8 to execute extrinsic apoptosis [47]. Instead, active caspase 8 cleaves a proapoptotic BH3-interacting domain death agonist (Bid) to form truncated Bid, which engages the mitochondria for the amplification and execution of death signals [47], as described in Section 2.1.1.

### 2.2. Formation of Hepatocyte ApopBDs

After hepatocytes commit to irreversible apoptosis, they form phosphatidylserine (PS)-rich, membrane-bound vesicles through cytoskeletal degradation, membrane blebbing, and cellular fragmentation [48]. These vesicles are known as hepatocyte ApopBDs. During the late execution phase of apoptosis, caspase-3 activation induces pyknosis, characterized by chromatin condensation and nuclear shrinkage [48]. This is followed by a loss of cell volume and cytoskeletal breakdown, causing the cell to shrink and the plasma membrane to bulge, resulting in membrane blebbing [49]. Subsequently, caspase-activated DNase triggers karyorrhexis, resulting in nuclear fragmentation with condensed chromatin [48]. Intact organelles are then condensed into small units and packaged within each bleb, except the nucleus, endoplasmic reticulum, and Golgi, which had already fragmented before being packaged in the blebs [48]. This implies that hepatocyte ApopBDs, which measure 0.5–5 µm in diameter, carry cell-specific cargoes, including nuclear fragments, metabolic enzymes, lipid droplets, oxidized lipids, aldehyde-protein adducts, and mitochondrial damage-associated molecular patterns, DAMPs. If hepatocytes are virally infected before apoptosis, ApopBDs may contain viral nucleic acids and proteins that function as pathogen-associated molecular patterns (PAMPs), which, upon internalization by resident non-parenchymal liver cells, amplify proinflammatory responses or profibrotic changes [50]. These recipient cells, which are typically located within the same organ as the apoptotic cells, possess receptors, including the T-cell Immunoglobulin and Mucin (TIM) family proteins, TAM, and stabilin receptors, that recognize PS on hepatocyte ApopBDs [51].

Although hepatocyte ApopBDs are classified as EVs, their structural characteristics, sizes, and biogenesis pathways differ from those of other types of EVs, such as exosomes and microvesicles. In disease contexts like non-alcoholic fatty liver diseases [7], alcoholic liver diseases, and hepatitis B virus, HBV,/hepatitis C virus, HCV-associated liver diseases [50,52,53], ApopBDs definitely play a role in disease progression.

## 3. Characteristics of Hepatocyte ApopBDs

To fully understand how hepatocyte ApopBDs communicate with other cells in the liver, it is important to be familiar with their characteristics. Thus, this section focuses on the structural and biochemical properties of hepatocyte ApopBDs and their role in liver injury.

### 3.1. Structural Features

Hepatocyte ApopBDs have distinctive morphological features that enable their identification and differentiation from other EVs [54]. A dying hepatocyte, typically 25–40 µm [55], disassembles into hepatocyte ApopBDs that range from approximately 0.5 to 5 µm in diameter [54]. This marked size variation reflects differences in the amount of cellular material incorporated during their formation from dying hepatocytes. Microscopic examination of hepatocytes undergoing apoptosis reveals distinct morphological changes associated with ApopBD formation [50]. Hepatocyte ApopBDs display an oval structure, reduced cell size, condensation and fragmentation of the nucleus, and the development of membrane-bound ApopBDs [50], which is different from the polygonal structure of a hepatocyte [48]. Electron microscopy provides enhanced visualization, highlighting characteristic features such as membrane envelopes that encase cytoplasmic and nuclear fragments of ApopBDs [48]. Although no specific in-depth analysis has been conducted to fully describe the structural and morphological characteristics of hepatocyte ApopBDs, it is generally believed that the ultrastructural features observed in ApopBDs, such as chromatin condensation and nuclear fragmentation, are similar across cell types.

### 3.2. Biochemistry of the Cargo Content

Hepatocyte ApopBDs are vesicles containing a highly diverse cargo that is reflective of the dying hepatocyte’s contents. These vesicles are vehicles for complex mixtures of proteins, lipids, nucleic acids, and intact organelles, such as mitochondria and ribosomes [50]. The composition of the cargo sometimes depends on the exposure of the dying hepatocytes. For instance, hepatocytes exposed to viruses such as HBV, HCV, and human immunodeficiency virus (HIV) will contain viral nucleic acids and proteins. Additionally, ApopBDs from hepatocytes exposed to ethanol will consist of lipid peroxides [50,56]. In other instances, the cargo contents of ApopBDs of the same exposure may be considerably different. This diversity in composition arises from both the random nature of membrane fragmentation during apoptosis and the regulated mechanisms that sort the cargo [57]. Therefore, prioritizing an omics-level investigation is essential to accurately identify the specific hepatocyte apoptotic cargo contents that trigger pathological changes in the liver.

Hepatocytes ubiquitously express metabolic enzymes such as cytochrome (Cyp) p450s, alcohol dehydrogenase, and aldehyde dehydrogenase [58], along with lipid metabolism proteins such as Apolipoprotein B and diacylglycerol acyltransferase, lipid droplets [59], and bile transporters like BSEP and MRP2 [60]. Consequently, the profiles of hepatocyte ApopBDs will differ significantly from those of other cells because they are expected to carry these metabolic signatures, which actively signal to KC and HSC upon internalization.

Given the unique roles of hepatocytes in the distribution of systemic proteins [61], we anticipate that intact ribosomes are likely to be predominant in hepatocyte ApopBDs. This feature will distinguish hepatocyte ApopBDs from those of non-hepatocytes, which typically contain fewer ribosomes. The presence of intact ribosomes and other organelles in hepatocyte ApopBDs demonstrates their capacity to transfer functional cellular elements to recipient cells.

Another outstanding feature of hepatocyte ApopBDs is the outer membrane of the ApopBDs. Hepatocytes express specific membrane marker proteins, including the asialoglycoprotein receptor, ASGPR [62], and SLC10A1 [63]. Since ApopBDs retain the molecular signature of the original cell, it is anticipated that hepatocyte ApopBDs will express these markers on their membranes. Whether these markers retain functional significance and contribute to liver pathogenesis remains an area requiring in-depth study. However, some of these markers are already associated with liver pathogenesis. For instance, SLC10A1, known as the HBV receptor [64], and ASGPR, which mediate the uptake of ApopBDs by surviving hepatocytes, also promote Severe Acute Respiratory Syndrome Coronavirus 2 infection of hepatocytes [65]. These surface proteins can facilitate interactions with recipient cells, induce viral persistence and inflammatory responses through mechanisms including direct binding, membrane fusion, and endocytosis-dependent uptake.

### 3.3. Phosphatidylserine Externalization

Under physiological conditions, PS resides in the inner leaflet of the plasma membrane, a distribution maintained by Adenosine Triphosphate (ATP)-dependent PS flippases [66]. However, during apoptosis, this asymmetry is disrupted, and PS is exposed on the outer surface of the membrane [67]. The externalization of PS, which makes it a critical “eat me” signal recognized by phagocytic receptors on adjacent cells, makes PS an essential biomarker for ApopBDs [68]. Hepatocytes do not normally express higher levels of PS than other cell types. However, they are more efficient at externalizing PS during apoptosis [69]. The rapid exposure of this PS implies that ApopBDs from hepatocytes will be quickly recognized and taken up by recipient cells. The PS signaling pathway is a key mechanism by which ApopBDs communicate their cargoes and influence the responses of recipient cells, particularly in liver damage and the resulting fibrogenic response.

### 3.4. Size Variations and Subpopulations

The broad range of sizes observed in hepatocyte ApopBDs encompasses both smaller vesicles resembling microvesicles and larger entities that contain substantial nuclear material [54]. This variation in size likely represents differences in the processes by which ApopBDs are formed across the various stages of hepatocyte apoptosis. In the early phases of apoptosis, smaller vesicles derived from the membrane may emerge [70], whereas later stages give rise to larger bodies containing condensed nuclear fragments and cytoplasmic organelles. The presence of these subgroups within the overall pool of ApopBDs suggests they may have distinct functional properties and cellular interactions, though a thorough characterization of functional differences based on size remains an area for future studies.

## 4. Intercellular Communications in the Liver

**Figure 2:**
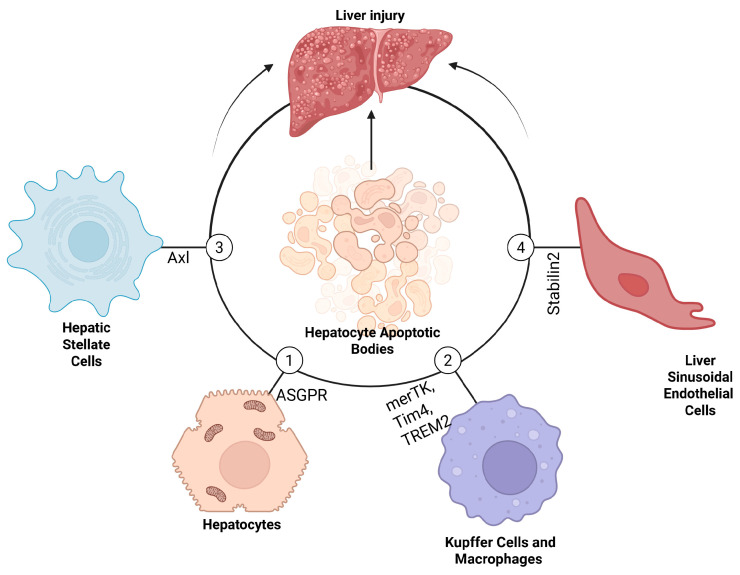
Intercellular communication of hepatocyte ApopBDs with liver resident cells. We observed from the review of the literature that: (1) Surviving hepatocytes use ASGPR for uptake of hepatocyte ApopBDs, which propagate death signals, proinflammatory miRNA 122, and lipotoxic cargoes to them. (2) KCs internalize hepatocyte ApopBDs through merTK, TIM4, and TREM2, which contain DNA CpG motifs that induce inflammatory changes via the NLRP3 pathway. This internalization also releases FasL and TNFα, perpetuating inflammation and hepatocyte apoptosis. (3) HSCs use the Axl receptors for the uptake of hepatocyte ApopBDs. Internalization of ApopBDs by HSCs has been observed. Hepatocyte-specific factors in ApopBDs elicit significant phenotypic changes in HSCs via the ROS-ERK1/2-IL-6 and JAK-STAT3 pathways. (4) Scavenger-receptor-rich liver sinusoidal endothelial cells internalize DAMPs and PAMPs-containing hepatocyte ApopBDs, which stimulate inflammatory changes. All these events described how the crosstalk between hepatocyte ApopBDs and liver resident cells contributes to liver injury.

The biogenesis and uptake of hepatocyte ApopBDs have biological implications for recipient cells. The molecules and organelles enclosed in these vesicles can drive changes in adjacent recipient cells, including hepatocytes, KCs, HSCs, and LSECs. A thorough understanding of the mechanisms of crosstalk between apoptotic hepatocytes and resident liver cells (summarized in Figure 2) will help us develop therapeutic strategies to mitigate liver disease progression.

### 4.1. Crosstalk with Surviving Hepatocytes

Hepatocyte-to-hepatocyte communication is a continuous process in both health and disease [71]. Paracrine signaling of metabolic stress-induced hepatocyte-derived trefoil factor 2 (TFF2), a hepatocyte-healing and fibrogenic protein, has been reported in the livers of individuals with steatohepatitis [72]. This supports a role for paracrine communication in perpetuating disease progression in the liver. While paracrine TFF2 signaling along the hepatocyte-to-hepatocyte axis may contribute to liver scarring, hepatocyte-derived meteorin-like, an antifibrotic protein, can reduce scar formation via hepatocyte-to-hepatocyte communication [73]. This substantiates that continuous crosstalk among hepatocytes is needed to maintain liver homeostasis.

A peculiar feature of liver damage is a constant cycle of hepatocyte apoptosis, which has been attributed to crosstalk between surviving hepatocytes and ApopBDs. Intercellular communication between hepatocyte ApopBDs and surviving hepatocytes via propagation of death signals has been documented [74]. Another piece of evidence of this crosstalk was substantiated by the uptake of HSC-derived EVs by hepatocytes, which altered their metabolic states [75]. Although hepatocytes lack the primary receptors for apoptotic body clearance, recent reports have documented the role of ASGPR on surviving hepatocytes for the uptake of hepatocyte ApopBDs [76]. microRNAs, such as proinflammatory miR-122, secreted by hepatocytes during apoptosis, are common components of hepatocyte ApopBDs. Internalization of miR-22 can turn on inflammatory responses in surviving hepatocytes [75].

The roles of hepatocyte ApopBDs in the metabolic reprogramming of surviving hepatocytes are another area of utmost importance. For example, the alteration of the metabolic state of surviving hepatocytes by ApopBDs from lipotoxic-stressed hepatocytes underlies the progression of fatty liver disease [77]. Hepatocyte apoptosis is usually accompanied by the generation of ROS during liver injury. This allows hepatocyte ApopBDs to convey ROS to surviving hepatocytes, thereby fostering an injury cycle in the liver [27].

### 4.2. Crosstalk with KCs and Macrophages

The liver, often referred to as a “graveyard,” is the primary site of clearance for apoptotic cells [78]. It is also a site where cellular waste is degraded, processed, and recycled under strictly immunologically controlled conditions [19]. As professional phagocytes, bone marrow-derived macrophages and liver-resident KCs efficiently perform these functions in the liver. KCs, the liver’s resident macrophages, are exceptionally effective phagocytes that clear apoptotic hepatocytes via PS-recognition receptors such as Mer tyrosine Kinase (TK) [79], TIM4 [80], and Triggering Receptor Expressed on Myeloid cells (TREM2) [81]. This activity, known as efferocytosis, is essential for maintaining homeostasis and averting inflammatory changes in the liver [82].

In a healthy liver, the removal of apoptotic hepatocytes is immunologically silent and does not elicit injurious outcomes. However, in a diseased liver, this seemingly benign process may become problematic and potentially lead to deleterious outcomes. Liver macrophages are functionally plastic, constantly changing their inflammatory phenotypes in response to signals from their microenvironment. Hepatocyte ApopBDs are one of the factors that provide strong signals that alter the proinflammatory-anti-inflammatory balance of liver macrophages. For example, internalization of hepatocyte ApopBDs by KCs triggers proinflammatory and pro-apoptotic signals [83].

Proinflammatory signals play a crucial role in coordinating immune responses by facilitating the recruitment of immune cells, including neutrophils, monocytes, and lymphocytes, to sites of inflammation or injury. While recruitment is vital for the resolution of benign hepatic conditions, failure to terminate this response may lead to a transition from protective mechanisms to chronic liver injury. Furthermore, the efferocytosis of hepatocyte ApopBDs is associated with the release of pro-apoptotic signals (i.e., FasL and TNF-α) [83], suggesting that the cycle of hepatocyte apoptosis and proinflammatory activation is likely to persist. Since ApopBDs are derived from damaged or virus-infected hepatocytes, their cargoes, which are either DAMPs (e.g., mitochondrial DNA, histones) or PAMPs (e.g., viral nucleic acid fragments or proteins), can activate the NLRP3 pathway [84,85], thereby triggering inflammatory responses.

It is unknown whether proinflammatory changes in KCs following internalization of hepatocyte ApopBDs are cargo-dependent, due to a lack of studies with comprehensive proteomic or molecular cargo analyses of hepatocyte ApopBDs specifically associated with KCs activation. However, it is well known that the uptake of hepatocyte ApopBDs by KCs activates inflammatory signaling. Further, proposed conceptual mechanisms involving cargo of ApopBDs is well supported. Hepatocyte ApopBDs, which are known carriers of CpG-rich DNA, trigger NF-κB activation and inflammatory responses when they interact with the Toll-like receptor 9 (TLR9) [86]. Moreover, lipid-rich hepatic ApopBDs have been implicated as triggers of proinflammatory cytokine release by KCs. Additionally, uptake of hepatocyte ApopBDs by macrophages contributes to cross-presentation of hepatocyte antigens, potentially linking apoptosis to autoimmune hepatitis [87].

### 4.3. Crosstalk with HSC

HSCs, like KCs, can engulf dying hepatocytes despite not being professional phagocytes. Due to their plasticity, HSCs can modify their functions in response to the liver’s microenvironment, thereby supporting liver homeostasis and repair [50,88]. While this function supports the already strained KCs during prolonged and excessive hepatocyte apoptosis, the uptake of hepatocyte ApopBDs may initiate fibrotic changes in HSCs [89]. This partly explains the correlation between hepatocyte apoptosis and liver fibrosis in liver failure [90]. The profibrotic response of HSCs to liver injury is intended for repair; however, when chronic damage persists, it results in excessive accumulation of extracellular matrix, leading to scar formation [91]. This disrupts the typical structure of the liver, eventually leading to hepatic dysfunction and potentially resulting in liver failure or hepatocellular carcinoma.

The internalization of hepatocyte ApopBDs activates HSCs through several interconnected signaling pathways. HSCs mainly engulf these ApopBDs via the Axl tyrosine kinase receptor. Our earlier study indicated that hepatocyte ApopBDs generated from HIV and alcohol exposure initiate profibrotic signaling pathways by generating reactive oxygen species (ROS) and interleukin-6, which subsequently activate profibrotic genes through the c-Jun N-terminal Kinase (JNK)- Extracellular Regulated Signal Kinases (ERK) 1/2 and Janus Kinase (JAK)- Signal Transducer and Activator of Transcription (STAT) 3 signaling pathways [50]. This activation, dependent on ROS, is a crucial process linking hepatocyte death to HSC activation.

More recently, we found that not all ApopBDs have an equal capacity to stimulate HSCs. Hepatocyte ApopBDs are more effective at triggering HSC activation than those derived from immune cells, such as lymphocytes [92]. This hepatocyte-specific response is driven by hepatocyte-derived growth factor, whose attenuation protected HSC from profibrotic activation [92]. This suggests that the mechanisms that drive these effects implicate the direct contents of the ApopBDs. Persistent activation of HSCs leads to the accumulation of excessive extracellular matrix, altering the metabolic state of hepatocytes and creating a vicious cycle that drives liver disease progression toward cirrhosis. The activation of HSCs by hepatocyte ApopBDs represents a fundamental mechanism in the pathogenesis of liver disease and highlights potential therapeutic targets for preventing the development of fibrosis.

### 4.4. Crosstalk with LSECs

There is evidence that LSECs can engulf hepatocyte ApopBDs to maintain vascular stability and reduce inflammation [93]. However, this interaction has garnered less focus than KCs and HSCs in the context of the clearance of hepatocyte ApopBDs. LSECs are some of the most endocytically active cells found in the body [94]. These endothelial cells possess high-affinity scavenger receptors, such as Scavenger Receptor A, B, B1, (CD) Cluster of Differentiation 34, and stabilins 1 and 2, which enable them to identify and internalize a diverse range of cellular debris, including hepatocyte ApopBDs [95]. It has been documented that LSECs take up hepatocyte ApopBDs due to their overall scavenging ability [71]. Once LSECs engulf hepatocyte ApopBDs, their functions are altered. However, the particular functional effects for LSECs after the uptake of hepatocyte ApopBDs are not as well characterized as the well-established mechanisms of KCs and HSCs activation [71].

Although no studies have clearly indicated that the uptake of hepatocyte ApopBDs is directly detrimental to LSECs, it is reasonable to consider that the internalization of these ApopBDs, particularly those carrying toxic viral proteins or other detrimental molecules (as observed in alcoholic, HBV or HCV-related liver damage) [71], might play a role in initiating or exacerbating the dysfunctions of LSECs via oxidative stress and proinflammatory induction, as well as inhibiting their endocytic potentials.

The contents of hepatocyte ApopBDs could lead to ROS generation in LSECs, which results in oxidative stress and subsequent cell damage [96]. Hepatocyte ApopBDs, which carry proinflammatory PAMPs and DAMPs may transport or trigger the release of proinflammatory substances that stimulate the inflammatory process seen in liver diseases [97].

Although LSECs effectively clear cellular debris, a persistent or excessive presence of hepatocyte ApopBDs, especially those originating from damaged or infected liver cells, might exceed the homeostatic capabilities of LSECs, resulting in functional deficiencies and aiding in liver disease progression.

## 5. Disease-Specific Roles of ApopBDs in Liver Pathophysiology

Hepatocyte apoptosis and the subsequent formation of ApopBDs constitute a key intersection at which various pathogenic pathways converge in liver disease. The uptake of ApopBDs by non-parenchymal cells is a primary mechanism driving both inflammation and fibrosis across a range of liver diseases caused by viral infections and alcohol misuse.

### 5.1. Hepatocyte ApopBDs in HBV Infection

HBV infection is characterized not only by direct viral replication in hepatocytes but also by complex interactions between infected cell death, intrahepatic phagocytes, and innate immune signaling. Hepatocyte apoptosis plays a central role in the balance between viral clearance and immune-mediated liver injury during HBV infection. As an intrinsic host defense mechanism, apoptosis eliminates infected hepatocytes, thereby restricting intracellular viral replication and limiting the spread of HBV to neighboring cells [98,99]. This process is often triggered by cytotoxic T lymphocytes (CTLs) and natural killer (NK) cells that recognize viral antigens presented on hepatocyte surfaces and secrete perforin, granzyme B, and FasL to induce programmed cell death of infected targets [100]. In this way, apoptosis functions as a non-inflammatory means of removing HBV-producing cells, contributing to viral control in acute infection. However, simultaneously, ApopBDs derived from infected hepatocytes can act as vesicular mediators that either enhance antiviral defense or promote viral persistence, depending on the efficiency of their clearance and detection by liver macrophages. This dual role underscores how apoptotic signaling can simultaneously aid HBV containment and drive inflammation-associated liver injury when dysregulated.

HBV-infected hepatocytes undergoing apoptosis release ApopBDs that encapsulate viral DNA, hepatitis B surface antigen (HBsAg), hepatitis B core antigen (HBcAg), and fragments of viral RNA [101,102]. These vesicles are efficiently recognized and engulfed by KCs, through apoptotic cell-recognition receptors such as MerTK and TIM4, among others [82,103]. Internalized viral nucleic acids within ApopBDs provide ligands for endosomal and cytosolic nucleic acid sensors, thereby linking apoptotic cell clearance to innate immune activation in the hepatic microenvironment [104,105,106]. To date, no study has directly demonstrated that HBV-infected hepatocyte-derived ApopBDs specifically deliver viral DNA to KCs, leading to activation of innate nucleic acid sensors. However, existing evidence supports the release of ApopBDs from HBV-infected hepatocytes, suggesting that these vesicles may expose neighboring non-parenchymal cells to viral components. Upon internalization, such vesicles could engage DNA and RNA-sensing pathways, thereby initiating innate immune responses.

Within hepatic macrophages, particularly KCs, HBV-derived DNA can engage endosomal TLR9, whereas viral DNA that gains access to the cytosol activates the cyclic GMP-AMP synthase (cGAS)-STING pathway [107,108,109]. Activation of these nucleic acid sensing pathways triggers downstream signaling through interferon regulatory factors and NF-κB, leading to the induction of interferon-β (IFN-β) and pro-inflammatory cytokines, including interleukin-6 (IL-6). The resulting cytokine milieu promotes antiviral states in neighboring hepatocytes and immune cells and can limit HBV replication, supporting a role for apoptotic body-mediated transfer of viral antigens and nucleic acids in innate antiviral defense within the hepatic microenvironment [110,111,112].

However, persistent or dysregulated activation of TLR9 and cGAS-STING signaling in KCs can become pathogenic. Sustained IFN-β and IL-6 production drives chronic hepatic inflammation, promotes recruitment and activation of additional immune cells, and exacerbates immune-mediated hepatocyte damage [113]. In the setting of chronic HBV infection, continuous generation of infected ApopBDs may therefore fuel a self-perpetuating cycle of apoptotic cell turnover, macrophage activation, and tissue injury that underlies progression to fibrosis and cirrhosis.

Environmental cofactors such as ethanol exposure can further modulate ApopBDs-macrophage interactions within the liver. Ethanol co-exposure promotes ApopBDs-formation in hepatocytes [50]. Simultaneously, it suppresses the expression of key efferocytosis receptors, including MerTK and TIM4, on hepatic macrophages, thereby impairing efficient recognition and clearance of apoptotic material [114]. Defective clearance can allow HBV-containing ApopBDs to accumulate and persist within the hepatic microenvironment, prolonging their engagement with innate immune sensors in KCs. Sustained nucleic acid sensing amplifies pro-inflammatory cytokine production, exacerbates hepatocellular injury, and promotes immune-mediated liver damage.

In addition to driving inflammation, HBV can exploit apoptotic vesicle pathways to evade immune surveillance. By packaging viral nucleic acids within ApopBDs or related vesicles, the virus may deliver its genome and transcripts to macrophages in a context that suppresses, rather than stimulates, type I interferon signaling. These vesicles have been shown to modulate macrophage activity toward a less antiviral phenotype, attenuating interferon responses that would otherwise limit viral replication [115,116]. Although direct evidence for ApopBDs functioning as immune-suppressive vehicles in HBV infection remains limited, several studies on viral EVs and infection-associated vesicle signaling support the plausibility of this mechanism [98,117]. Through this strategy, HBV appears to co-opt physiological efferocytosis pathways normally responsible for the silent removal of apoptotic cells to establish a tolerogenic and blunted innate immune environment that promotes viral persistence.

Collectively, these findings identify ApopBDs derived from HBV-infected hepatocytes as pivotal mediators in the hepatic response to infection, functioning at the intersection of cell death, innate immune sensing, inflammatory amplification, and viral immune evasion. Therapeutic interventions that restore efficient ApopBDs clearance and maintain appropriate expression of efferocytosis receptors such as MerTK and TIM4, particularly under conditions of ethanol exposure, may strengthen antiviral immunity in chronic HBV infection. Furthermore, precise modulation of TLRs and cGAS-STING signaling could mitigate excessive innate activation, thereby reducing immune-mediated hepatic injury while preserving effective antiviral defense.

### 5.2. Hepatocyte ApopBDs in HCV Infection

As in other liver diseases, hepatocyte apoptosis and the uptake of ApopBDs by non-parenchymal cells play significant roles in the pathogenesis of HCV-related liver conditions, including inflammation and fibrosis [118]. HCV-infected hepatocytes are highly susceptible to apoptosis because HCV upregulates caspases 8, 9, and 3 [119]. Moreover, HCV activates ROS production, the NLRP3 inflammasome, and the IL-1β inflammasome pathway, thereby contributing to hepatocyte apoptosis [120,121].

The positive correlation between fibrotic progression and Fas/FasL expression in liver biopsies of individuals infected with HCV confirms the roles of extrinsic apoptosis in the progression of HCV-associated liver damage [122]. In fact, the critical roles of hepatocyte ApopBDs in HCV infection are so significant that apoptotic markers have emerged as essential diagnostic tools [123].

The key features of ApopBD formation in HCV-infected hepatocytes are similar to those previously described in Section 2, except that HCV proteins are included in the cargo [118]. The phenotypic modification of KCs and HSCs upon internalization of HCV-laden ApopBDs supports the role of ApopBDs in driving HCV liver disease [118]. Specifically, ApopBDs derived from HCV-infected hepatocytes drive the release of proinflammatory cytokines, including IL-1β, TNF-α, and IL-6, thereby creating an inflammatory microenvironment that exacerbates liver injury [124]. Transforming growth factor (TGF)-β, which sensitizes HSCs to fibrotic changes, has been identified as an essential component of HCV-infected hepatocyte ApopBDs, leading to extracellular matrix deposition and liver scarring, a key feature of HCV infection [125].

Since HCV replication stimulates ROS production, hepatocyte ApopBDs in the context of HCV definitely contain ROS and oxidative products. ROS within the HCV-derived hepatocyte ApopBDs can serve as a self-amplifying trigger of liver injury [121]. Given the infectious nature of HCV, this can bolster the continuous generation of injurious ApopBDs, ultimately connecting HCV infection to chronic liver disease and hepatocellular carcinoma. This was validated by studies that correlated hepatocyte apoptosis with the severity of fibrosis in individuals infected with HCV [126].

Although active HCV is likely cleared during hepatocyte apoptosis, HCV proteins, which are likely packaged into ApopBDs, are anti-apoptotic. This suggests that while HCV infection triggers a cascade of hepatocyte apoptosis, leaving behind anti-apoptotic proteins that activate survival pathways [127] will either perpetuate the infection cycle or set the stage for hepatocellular carcinogenesis. Previous studies have documented that the internalization of HCV-infected hepatocyte ApopBDs triggers the release of STAT3 and nuclear factor(NF)-κB, which enhances hepatocyte survival and proliferation pathways [124]. This enigmatic shift from proapoptotic to anti-apoptotic responses in hepatocytes seems to contribute to hepatocellular carcinoma in the setting of HCV. In HCV infection, ApopBDs can be pro-viral, acting as carriers of infectious material that spreads infection, a process that is significantly potentiated by second hits such as alcohol exposure [98]. Remarkably, in HBV-infection, hepatocyte apoptosis (and likely, ApopBDs packaging/clearance) can be antiviral by restricting the release of this virus [128]

While the role of ApopBDs in regulating antiviral defense remains unclear, it is known that apoptosis often suppresses IFN pathways, limiting ApopBD-driven antiviral sensing unless paired with strong pattern-recognition receptor (PRR) triggers [129]. In addition, ApopBDs can affect immune activation potential by delivering antigen for cross-presentation, which in the liver supports either protective CD8 priming or tolerance/exhaustion, depending on the context [129].

### 5.3. Hepatocyte ApopBDs in Alcohol-Associated Liver Disease (ALD)

Chronic alcohol intake induces profound metabolic and oxidative stress in hepatocytes through CYP P450 2E1-mediated ROS generation, ER stress, and mitochondrial dysfunction [130,131]. These processes lead to extensive apoptosis and a massive accumulation of hepatocyte ApopBDs. Alcohol downregulates key efferocytic receptors, such as MerTK, TIM4, and stabilin-2, on KCs, compromising ApopBDs removal [132]. Uncleared hepatocyte ApopBDs undergo secondary necrosis, releasing mitochondrial DNA, High Mobility Group Box 1, ROS, and extracellular ATP, all potent DAMPs known to activate TLR9, NLRP3, and gasdermin-mediated pyroptosis [133,134]. The chronic necro-inflammatory state arising from defective clearance is the central pathogenic axis in alcoholic hepatitis. Increasing evidence shows that hepatocyte ApopBDs enriched in oxidized lipids can directly activate KC inflammasomes [135]. In ALD, the inflammasome perpetuates IL-1β signaling, further enhances hepatocyte apoptosis, and HSCs are key events in fibrogenesis [136,137]. In alcoholic hepatitis, hepatocytes aberrantly engulf ApopBDs through ASGPR and αvβ integrins, a process linked to intracellular oxidative bursts and Mitogen-Activated Protein Kinase (MAPK) activation [138]. This “bystander apoptosis” mechanism expands zones of necrosis and promotes lesion progression, echoing the notion that apoptotic signaling magnifies liver injury even beyond the primary insult [139]. Bystander apoptosis refers to programmed cell death in cells that are not directly targeted by the original insult but die in response to signals released by nearby injured, infected, or immune-activated cells.

### 5.4. Hepatocyte ApopBDs in Metabolic Dysfunction–Associated Steatohepatitis (MASLD/NASH)

NASH represents a lipotoxic environment in which saturated fatty acids, ceramides, and cholesterol induce caspase-9 and caspase-3-mediated apoptosis [140,141]. Therefore, hepatocyte ApopBDs in NASH carry unique cargo enriched in oxidized lipids, cholesterol crystals, miRNAs (i.e., miR-122, miR-192), and bioactive ceramides. The KC uptake of lipotoxic hepatocyte ApopBDs via CD36 and integrins drives NLRP3 activation, IL-1β release, and sustained necro-inflammation [142]. As previously noted, this inflammatory milieu is central to fibrotic progression [143]. Hepatocyte ApopBDs directly deliver miR-122, miR-192, and oxidized lipids to HSCs, thereby promoting TGF-β signaling, ROS production, and fibrogenic transcriptional reprogramming, a mechanism that aligns with HSC activation models [133,143]. Sheddase ADAM17 is elevated in NASH and cleaves MerTK, hindering apoptotic body clearance [144]. The resulting accumulation of uncleared ApopBDs perpetuates inflammation, mirroring mechanisms seen in ALD but driven by lipotoxicity rather than ethanol. Theoretically, ApopBDs release can help in distinguishing disease activity and stage because hepatocyte apoptosis increases with steatohepatitis and progressive fibrosis. However, in the literature, other biomarkers, such as cytokeratin 19 (SK-18) or small EVs, are cited as “apoptosis signatures” [145,146,147]. Since studies have not been conducted on purified ApopBDs, these markers can be interpreted as “ApopBDs-adjacent” signals.

### 5.5. Hepatocyte ApopBDs in Other Injury and Regenerative Contexts

#### 5.5.1. Drug-Induced Liver Injury

Massive hepatocyte apoptosis characterizes many forms of drug-induced liver injury, and hepatocyte ApopBDs function as antigenic sources that activate dendritic cells and adaptive immune pathways [148]. By releasing liver-enriched miRNAs and neoantigens, hepatocyte ApopBDs may contribute to chronicity in idiosyncratic drug-induced liver injury.

#### 5.5.2. Regeneration After Hepatectomy

Conversely, the clearance of hepatocyte ApopBDs by LSECs and KCs supports regenerative signaling. Uptake of ApopBDs by hepatocytes stimulates hepatocyte growth factor, Wingless-related integration-1 (Wnt) ligands, and endothelial remodeling, processes essential for hepatocyte proliferation [149,150]. This beneficial facet of the biology of hepatocyte ApopBDs underscores that apoptosis is not solely destructive but can also orchestrate regenerative programs when properly regulated. However, as this review aims to focus on the role of hepatocyte ApopBDs as contributors to liver diseases, we will refrain from exploring their benefits in greater detail.

## 6. Therapeutic and Diagnostic Implications

Beyond intercellular messengers, hepatocyte ApopBDs, are now recognized as active mediators of hepatic inflammation, fibrogenesis, and intercellular communication. Increase in evidence has shown that they contain mitochondrial DNA, nuclear fragments, oxidized lipids, and microRNAs which provide mechanistic links between hepatocyte apoptosis and the propagation of injury to neighboring KCs, HSCs, and LSECs. Accordingly, therapeutic targeting of hepatocyte ApopBDs signaling and the development of Hepatocyte ApopBDs-derived biomarkers represent emerging frontiers in liver disease management. The therapeutic and diagnostic implications are summarized in Table 1.

### 6.1. Therapeutic Implications

#### 6.1.1. Targeting MerTK-Mediated Pro-Inflammatory Signaling

When it comes to several engulfment receptors implicated in hepatocyte ApopBDs’ clearance, MerTK plays a dominant role in shaping hepatic inflammatory and fibrogenic responses. When cells are in homeostatic states, MerTK activates phagocytosis and anti-inflammatory cytokine production (IL-10, TGF-β) through PI3K–AKT and STAT3 signaling [151]. However, chronic liver disease drives persistent hepatocyte apoptosis, which leads to enhanced MerTK activation in KCs and HSCs and ultimately promotes TGF-β1 production, extracellular matrix deposition, and scar progression [152].

In experimental models, MerTK inhibition using small molecules or soluble decoy receptors, has been shown to attenuate ApopBDs-mediated activation of HSCs, reduce collagen synthesis, and decrease fibrosis progression [151,153]. These findings highlight MerTK as a compelling therapeutic target in (NASH), alcoholic steatohepatitis, and chronic viral hepatitis.

#### 6.1.2. Modulating TIM4 and Stabilin-2 to Reduce Pathological Efferocytosis

TIM4 and stabilin-2 are major (PS) receptors expressed on KCs and LSECs. While their normal role is to maintain hepatic immune tolerance by clearing apoptotic debris, chronic injury environments result in dysregulated efferocytosis, leading to repeated TGF-β release and increase inflammation [144]. Therapeutic modulation of TIM4 or stabilin-2 via monoclonal antibodies or blocking peptides represents another potential strategy to interrupt cycles of inflammation and fibrogenesis.

#### 6.1.3. Targeting Hepatocyte–Hepatocyte Propagation of Cell Death

Hepatocytes can internalize ApopBDs via ASGPR, integrins, and CD91/Low-density lipoprotein receptor-related protein -1. In alcohol-associated liver disease, ASGPR-mediated uptake of apoptosis-derived material promotes oxidative stress, mitochondrial permeabilization, and secondary apoptosis [134]. Pharmacologic modulation of ASGPR, already used in GalNAc-siRNA delivery systems may offer a means to reduce intracellular propagation of apoptotic signals. Additionally, inhibition of bridging molecules such as Gas6 and MFG-E8 that connect ApopBDs to hepatocyte integrins may also reduce apoptotic amplification.

#### 6.1.4. Reducing ApopBDs Biogenesis and Lipotoxic Death

Several therapeutic strategies aim to limit ApopBDs formation at the source by targeting upstream triggers of hepatocyte apoptosis including antioxidants and ROS scavengers which reduce mitochondrial oxidative stress and caspase activation [154], Caspase inhibitors also attenuate hepatocyte apoptosis in models of NASH and viral hepatitis [152] while lipotoxicity inhibitors, such as Acetyl-CoA Carboxylase or diacylglycerol O-acyltransferase 2 blockers, reduce lipid-driven hepatocyte apoptosis and downstream ApopBDs generation. By reducing the release of ApopBDs at their inception, these approaches prevent downstream activation of macrophages and stellate cells.

#### 6.1.5. Interrupting HSC Activation via ApopBDs Cargo

ApopBDs derived from injured hepatocytes are enriched with mitochondrial DNA and oxidized lipids that activate TLR9, NF-κB, and MAPK pathways in HSCs [155]. Therapeutic strategies include TLR9 antagonists, which prevent mtDNA-driven stellate cell activation and MAPK inhibitors, which limit JNK/p38 signaling and reduce fibrogenesis. Importantly, HSC apoptosis can release ApopBDs carrying anti-fibrotic miRNAs such as miR-29 and miR-19b [156]. Enhancing such beneficial ApopBDs signaling potential via engineered vesicles may represent a future antifibrotic strategy.

### 6.2. Diagnostic Implications

#### 6.2.1. Circulating ApopBDs Quantification as a Marker of Hepatocyte Apoptosis

Levels of circulating ApopBDs increase markedly in conditions characterized by prominent hepatocyte apoptosis, including NAFLD/NASH, alcoholic liver disease, HBV/HCV infection, and drug-induced liver injury [5,153]. Their enumeration by flow cytometry or nanoparticle tracking analysis can complement transaminase tests, providing a more direct measure of programmed cell death burden. Unlike alanine aminotransferase or aspartate aminotransferase, which reflect membrane leakage ApopBDs reflect regulated apoptosis, offering mechanistic specificity.

#### 6.2.2. ApopBDs-Associated DNA and RNA as Molecular Biomarkers

ApopBDs carry Mitochondrial DNA which is known as activator of TLR9 and marker of neuroinflammatory death [155] and microRNAs, including miR-122, miR-34a, miR-29, and miR-19b, which correlate with fibrosis severity, apoptosis rate, and metabolic stress [156]. Circulating ApopBDs-derived mtDNA and miRNAs provide a non-invasive molecular fingerprint of ongoing hepatocellular injury and fibrogenesis.

#### 6.2.3. Surface Markers and Receptor–Ligand Signatures

ApopBDs express distinct surface molecules depending on the mode of hepatocyte injury: Calreticulin (another “eat-me” signal) marks ER-stress-induced apoptosis [149], Desialylated glycoproteins are enriched in alcohol-related apoptosis [134], Oxidized phospholipids and complement fragments identify inflammation-induced apoptosis [144]. These features may have diagnostic value in distinguishing ALD from NASH or viral hepatitis.

### 6.3. ApopBDs Profiling for Disease Staging and Treatment Response

As biomarkers, ApopBDs possess several advantages, such as rising early in disease progression, preceding fibrosis; also contain cargo that reflects the mode of cell death (apoptosis, necroptosis, pyroptosis) [157] and their levels reduce with successful treatment, providing a dynamic measure of therapeutic response. This makes ApopBDs profiling well-suited for clinical monitoring in NASH drug trials, antiviral therapy in HBV/HCV, and interventions targeting alcoholic steatohepatitis.

**Table 1:** Therapeutic and diagnostic strategies leveraging hepatocyte apoptotic body (ApopBDs) biology in liver disease.

Target	Biological Rationale	Proposed Intervention	Potential Clinical Utility	Key References
MerTK signaling in KCs and HSCs	Chronic ApoBD clearance via MerTK promotes TGF-β–driven inflammation and fibrogenesis	MerTK small-molecule inhibitors; soluble MerTK decoy receptors	Attenuation of macrophage- and stellate-cell–mediated fibrosis in NASH, ASH, and chronic viral hepatitis	[150,151]
TIM4 and Stabilin-2–mediated efferocytosis	Dysregulated phosphatidylserine-dependent efferocytosis sustains inflammatory signaling	Monoclonal antibodies or blocking peptides targeting TIM4 or Stabilin-2	Reduction in pathological efferocytosis and interruption of chronic inflammatory cycles	[133]
Hepatocyte ApoBD uptake pathways (ASGPR, integrins, CD91/LRP1)	ApoBD internalization propagates oxidative stress and secondary hepatocyte apoptosis	Pharmacologic modulation of ASGPR; inhibition of bridging molecules (Gas6, MFG-E8)	Limitation of hepatocyte-to-hepatocyte amplification of apoptotic signaling	[153]
ApoBD biogenesis at the source	Excessive hepatocyte apoptosis increases ApoBD burden and downstream immune activation	Antioxidants, ROS scavengers; caspase inhibitors; lipotoxicity inhibitors (ACC, DGAT2 blockers)	Upstream suppression of ApoBD release and downstream macrophage/HSC activation	[134,151]
ApoBD cargo–mediated HSC activation	ApoBD-associated mtDNA and oxidized lipids activate TLR9, NF-κB, and MAPK pathways in HSCs	TLR9 antagonists; MAPK (JNK/p38) inhibitors	Direct inhibition of ApoBD-driven stellate cell activation and fibrogenesis	[154,155]
Engineered ApoBDs carrying antifibrotic miRNAs	Certain ApoBDs contain miRNAs (e.g., miR-29, miR-19b) with antifibrotic effects	Engineered or enriched vesicles delivering antifibrotic miRNAs	Novel antifibrotic therapeutic platforms targeting stellate cells	[5,152]
Circulating ApoBD quantification	ApoBD levels reflect regulated hepatocyte apoptosis rather than nonspecific injury	Flow cytometry; nanoparticle tracking analysis	Non-invasive assessment of hepatocyte apoptosis burden across liver diseases	[154,155]
ApoBD-associated mtDNA and miRNAs	ApoBD cargo mirrors molecular pathways of injury and fibrosis	Liquid-biopsy profiling of mtDNA and apoptosis-associated miRNAs	Molecular biomarkers for fibrosis severity, metabolic stress, and inflammatory activity	[133,148]
ApoBD surface markers	Injury-specific surface signatures distinguish apoptosis etiologies	Detection of calreticulin, desialylated glycoproteins, oxidized phospholipids	Etiology-specific discrimination (ALD vs. NASH vs. viral hepatitis)	[153]
ApoBD profiling for disease staging and response	ApoBD levels rise early and decline with effective therapy	Longitudinal ApoBD monitoring	Early disease staging and dynamic assessment of therapeutic response	[156]

## 7. Conclusions

Hepatocyte apoptosis, which leads to the formation of ApopBDs, is a key feature of the onset and progression of liver diseases such as MASLD/NASH, ALD, and virus-associated liver disease. While hepatocyte apoptosis is the central mechanism of the onset of liver injury, intercellular crosstalk between hepatocyte ApopBDs and resident liver cells contributes to the progression of liver damage. Surviving hepatocytes use ASGPR to internalize apoptotic hepatocyte bodies that propagate proinflammatory cytokines and death signals, thereby sustaining inflammation and the cycle of hepatocyte apoptosis. KCs, professional phagocytes, are primed for inflammatory responses after uptake of hepatocyte ApopBDs via the MerTK, Tim4, and TREM2 receptors. Upon recognition of ROS-containing hepatocyte ApopBDs by Axl, the bodies are internalized by HSCs, which are subsequently stimulated to undergo profibrotic activation via the JNK-ERK1/2 and JAK-STAT3 pathways. Liver sinusoidal endothelial cells also contribute to this crosstalk by internalizing hepatocyte ApopBDs, thereby inducing inflammatory changes and impairment of their scavenging capacity.

Additionally, hepatocyte ApopBDs, which carry specific hepatocyte signatures, are generated early in liver disease and can function as diagnostic tools because of their presence in liver interstitial fluid and circulation. Targeting hepatocyte apoptosis and ApopBDs formation are critical therapeutic options for improving liver injury.

## Data Availability

No new data were created or analyzed in this study.

## Abbreviations

The following abbreviations are used in this manuscript:

ApopBDs	Apoptotic bodies
EVs	Extracellular vesicles
KCs	Kupffer cells
HSC	Hepatic stellate cells
Bcl2	B-cell lymphoma 2
TNFR	Tumor Necrosis Factor Receptor
TRAIL	Tumor Necrosis Factor-Related Apoptosis-Inducing Ligand
FasL	Fas Ligand
TNFα	Tumor Necrosis Factor alpha
PS	Phosphatidylserine
PAMPs	Pathogen-Associated Molecular Patterns
DAMPs	Damage-Associated Molecular Patterns
HBV	Hepatitis B virus
HCV	Hepatitis C virus
HIV	Human immunodeficiency virus
ASGPR	Asialoglycoprotein receptor
JAK	Janus Kinase
STAT	Signal Transducer and Activator of Transcription
JNK	c-Jun N-terminal kinase
ERK	Extracellular signal-regulated kinase
Wnt	Wingless-Type MMTV Integration Site Family, Member
NF	Nuclear Factor
CTLs	Cytotoxic T Lymphocytes
NK	Natural Killer
ATP	Adenosine Triphosphate
NLRP3	Nod-like receptor family pyrin domain containing 3
MAPK	Mitogen-Activated Protein Kinase
NASH	Non-Alcoholic Steatohepatitis
TGF	Transforming Growth Factor
TK	Tyrosine Kinase
TREM2	Triggering Receptor Expressed on Myeloid Cells
Tim	T cell immunoglobulin and mucin domain
